# Identification of *COX4I2* as a hypoxia-associated gene acting through FGF1 to promote EMT and angiogenesis in CRC

**DOI:** 10.1186/s11658-022-00380-2

**Published:** 2022-09-05

**Authors:** Jie-pin Li, Yuan-jie Liu, Shu-hong Zeng, Hai-jian Gao, Yu-gen Chen, Xi Zou

**Affiliations:** 1 Zhangjiagang TCM Hospital Affiliated to Nanjing University of Chinese Medicine, Zhangjiagang, 215600 Jiangsu China; 2 Affiliated Hospital of Nanjing University of Chinese Medicine, Jiangsu Province Hospital of Chinese Medicine, Nanjing, 210029 Jiangsu China; 3 No. 1 Clinical Medical College, Nanjing University of Chinese Medicine, Nanjing, 210023 Jiangsu China; 4 Jiangsu Collaborative Innovation Center of Traditional Chinese Medicine in Prevention and Treatment of Tumor, Nanjing University of Chinese Medicine, Nanjing, 210023 China

**Keywords:** Colorectal cancer, *COX4I2*, Fibroblast growth factor 1, Epithelial–mesenchymal transition, Angiogenesis, Cancer-associated fibroblasts

## Abstract

**Background:**

Current evidence suggests that the hypoxic tumor microenvironment further aggravates tumor progression, leading to poor therapeutic outcomes. There is as yet no biomarker capable of evaluating the hypoxic state of the tumor. The cytochrome* c* oxidase (COX) subunit is crucial to the mitochondrial respiratory chain.

**Methods:**

We investigated the potential oncogenic role of COX subunit 4 isoform 2 gene (*COX4I2*) in colorectal cancer (CRC) by least absolute shrinkage and selection operator (LASSO) and COX regression analysis to examine whether *COX4I2* overexpression can predict colorectal cancer (CRC) prognosis. The association of *COX4I2* levels with clinical features and its biological actions were evaluated both in vitro and in vivo.

**Results:**

Our analysis showed that elevated *COX4I2* levels were correlated with poor clinical outcomes. We also observed that that *COX4I2* may be involved in epithelial-mesenchymal transition, activation of cancer-related fibroblasts and angiogenesis in relation to fibroblast growth factor 1.

**Conclusions:**

The *COX4I2* level may be a predictor of outcome in CRC and may represent a novel target for treatment development.

**Graphical Abstract:**

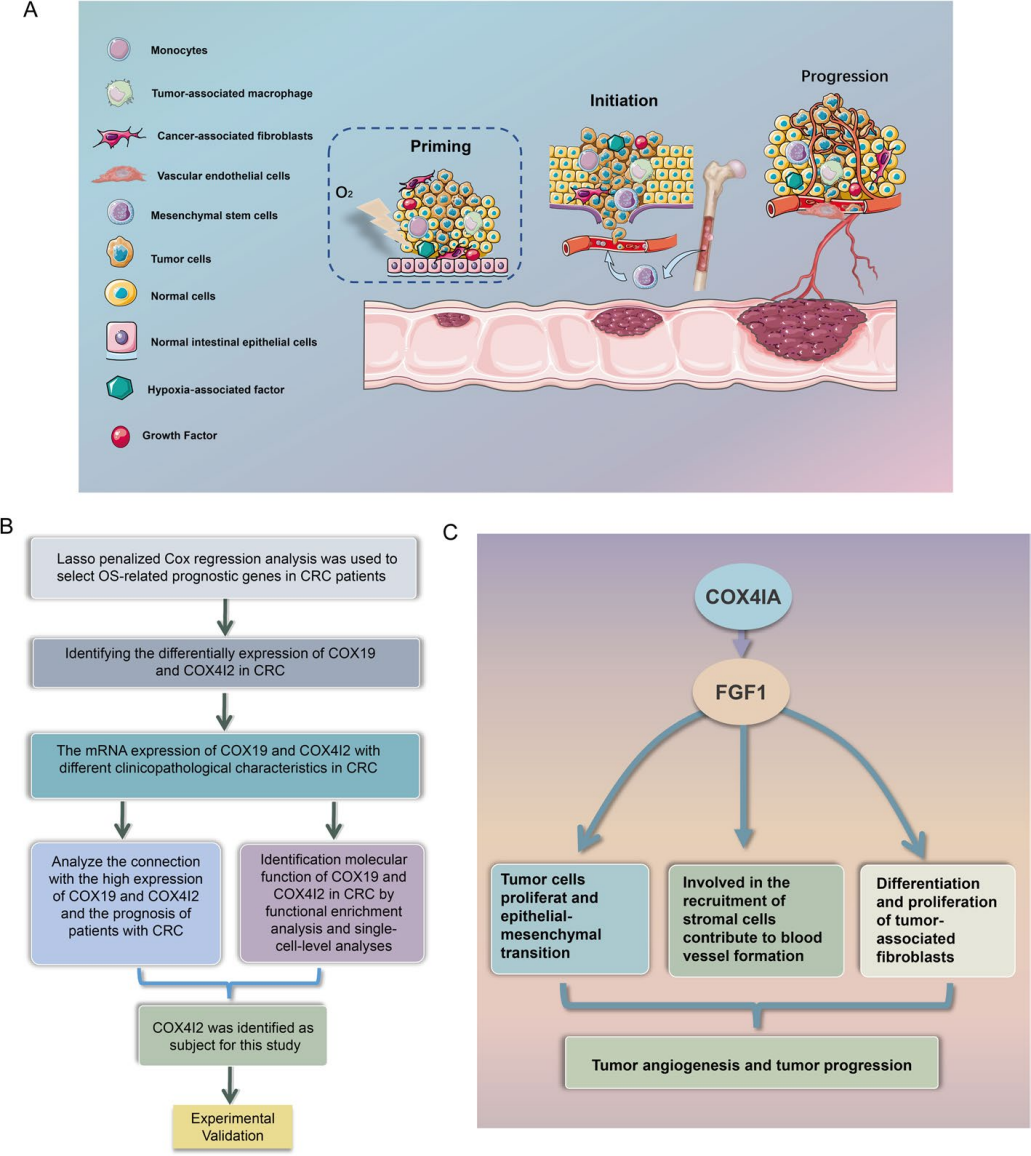

**Supplementary Information:**

The online version contains supplementary material available at 10.1186/s11658-022-00380-2.

## Introduction

Colorectal cancer (CRC) is a common digestive neoplasm, ranking third in incidence among malignancies and second in lethality among cancers [1]. Its pathogenesis is complex, involving numerous predisposing factors [2–4], and has been linked to genetics, diet, and lifestyle habits. Statistics show that 30–50% of CRC tumors recur with distant metastases after surgery, especially to the liver, lung, bone and ovaries, and 5-year survival often does not exceed 10% [5].

Tumor growth and metastasis require vascular nourishment, and tumor neovascularization is a feature of the tumor microenvironment [6]. The hypoxic microenvironment disrupts the balance of vascular-related factors, activates endothelial cells, and increases vascular permeability, thereby promoting the delivery of nutrients to cancer cells to enhance migration and proliferation, resulting in tumor progression [7, 8]. In recent years, anti-angiogenic therapy, such as bevacizumab and regorafenib, has been widely used in the treatment of CRC [9, 10]. However, the formation of tumor-associated blood vessels involves the action of multiple genes and signaling pathways, with wide-ranging and complex regulatory mechanisms, and relatively few patients can benefit from anti-angiogenic therapy. Thus, it is particularly important to screen for suitable target populations and prognostic markers associated with anti-angiogenic therapy.

Cytochrome* c* oxidase (COX) is the last enzyme in the respiratory chain and is responsible for the catalytic transfer of electrons to oxygen (O_2_) [11]. The mitochondrial COX gene (*COX*) encodes the catalytic subunit that is complexed with nuclear gene-encoded structural subunits [12]. Subunit 4 (COX4) is the largest of these and is involved in the mitochondrial respiratory chain, optimizing respiratory efficiency at different oxygen concentrations. As a result, mammalian cells respond to hypoxic environments by altering the composition of the COX subunits [13]. Levels of the protein-coding genes *COX4I1* and *COX4I2* are regulated by oxygen concentration: under normal concentrations, *COX4I1* levels rise, while those of *COX4I2* decrease. In hypoxic conditions, hypoxia-inducible factor (HIF) induces the expression of *COX4I2*, thereby raising *COX4I2* levels, altering the membrane potential of the mitochondrion and elevating levels of ATP and reactive oxygen species (ROS) [14]. ROS levels have been linked to angiogenesis, including activities such as stimulating growth and migration in endothelial cells and modulating the levels of vascular endothelial growth factor (VEGF), its receptor, nuclear factor kappa-B (NF-κB), mitogen-activated protein kinase (MAPK), and matrix metalloproteinases (MMPs) [15–17].

It is well-known that hypoxia is related to tumor progression and poor prognosis. However, the action of *COX4I2*, which is upregulated under hypoxic conditions, in tumor progression is poorly understood. Here, we demonstrated a novel molecular pathway whereby activation of *COX4I2* significantly induces fibroblast growth factor 1 (FGF1) expression and thus promotes tumor-associated angiogenesis and the activation of cancer-associated fibroblasts (CAFs).

## Materials and methods

The workflow and mechanism diagram of this study is provided in the Pictorial Abstract.

### Reagents and antibodies

Information on the reagents and antibodies is given in Additional file 1: Table S1. The concentrations used were based on previous studies, as recommended in the protocols provided. Further details are given in the Additional file.

### Cell culture

The human CRC cell lines SW620 (Cat. no. TCHu101), SW480 (Cat. no. SCSP-5033), RKO (Cat. no. TCHu117), HCT116 (Cat. no CL-0096), HT-29 (Cat. no. TCHu103), and LoVo (Cat. no. SCSP-514) were acquired from the cell bank of the Chinese Academy of Sciences (Shanghai, China). The human normal colon epithelial cell (NCM460) (Cat no. CP-H040), human mesenchymal stromal cells (MSCs) (Cat. no. CP-H166), and vascular endothelial cells (human umbilical vein endothelial cells [HUVECs]; Cat. no. CL-0122) were obtained from Cyagen Biosciences (Guangzhou, China). SW620 cells were grown in Leibovitz’s L-15 medium with 10% fetal bovine serum (FBS), while the other lines were grown in Dulbecco’s Modified Eagle Medium (DMEM) supplemented with 10% FBS under 5% CO_2_ at 37 °C. All media were supplemented with 1% penicillin/streptomycin. Cells passed regular mycoplasma contamination testing. All cells were recently authenticated by short tandem repeat (STR) profiling.

### Ethics and sample collection

Thirty paired CRC tissues and adjoining normal tissues (margin: 5 cm) were obtained intra-operatively from untreated patients at the Jiangsu Province Hospital of Chinese Medicine. All experiments were conducted in accordance with the Declaration of Helsinki and in accordance with the relevant designated guidelines and regulations [18]. Tumors were scored using the 8th edition of the American Joint Committee on Cancer tumor-node-metastasis (TNM) system [19]. The samples were rinsed with cold phosphate buffered saline, quick-frozen in liquid nitrogen, and were stored at − 80 °C until analysis. Serum samples obtained preoperatively were stored at – 80 °C.

### Immunohistochemistry

Immunohistochemistry (IHC) was performed according to a previously published protocol [20]. Micrographs were obtained using a Nikon Eclipse Ni-E microscope (Nikon Corp., Tokyo, Japan) (original magnification: ×400). The H-SCORE (range: 0–300, with higher scores indicating stronger positive staining) was determined according to the published protocol.

### Establishment of hypoxia model

A stock solution of CoCl_2_ was prepared by dissolving solid CoCl_2_ in serum-free DMEM. Using a previously published method [21], we pretreated cells with 300 µmol/L CoCl_2_ for 30 h. CoCl_2_ induces hypoxia through blocking the degradation of HIF-α, thereby activating the hypoxia cascade [22].

### Western blotting

The western blotting method is described in [23]. The protein bands (including β-actin as the loading control) were visualized with a gel imaging system (ChemiDoc XRS+; Bio-Rad Laboratories, Hercules, CA, USA), and relative concentrations were determined.

### RNA interference and overexpression of plasmid construction and transfection

SW480 and RKO cells, both of which express relatively high levels of COX4I2, were selected for subsequent experiments. The plasmids described here were all constructed by the commercial hi-tech company GeneChem (Shanghai, China). The procedures for constructing overexpression and RNA interference (RNAi) plasmids are described in Additional file. Three short hairpin interfering RNAs (shRNAs) targeting COX4I2 were constructed, and the one with the highest inhibitory efficiency was selected for subsequent experiments. The shRNA-COX4I2 plasmid (sh-COX4I2), a control non-targeting plasmid (NC), and the overexpressing plasmid (oe-COX4I2) were transfected into 70%-confluent cells using Lipofectamine 3000, in accordance with the described protocol. Overexpression, knockdown, and transfection efficiency were evaluated using western blotting and green fluorescent protein (GFP) expression.

### Colony-formation assay

Colony-forming assays were conducted according to published protocols [24]. Colony numbers were determined under a compound light microscope (model BX53; Olympus Corp., Tokyo, Japan).

### Cell Counting Kit-8 assay

Viability was assessed with a Cell Counting Kit-8 (CCK8) assay kit following published methodology [25]. Cells were grown in 96-well plates (5 × 10^3^ cells/well) in 100 μL serum-free medium for 12 or 24 h at 37 °C. CCK-8 solution (100 μL/well) was added for 2 h, following which the cells were grown for 12 or 24 h and absorbance read at 450 nm using a microplate reader (Synergy HT; Biotek Instruments, Winooski, VT, USA ).

### Rescue experiment

For *COX4I2* overexpression rescue experiments, the *COX4I2*-overexpressing cell lines were pretreated with 5 μM PD166866 for 12 h. PD166866 is a synthetic small molecule that inhibits the FGF-1 receptor tyrosine kinase (FGFR1); it is highly selective for FGFR1 with potential use as anti-proliferative/antiangiogenic agent for such therapeutic targets as tumor growth and neovascularization [26, 27]. Optimal concentration selection of PD166866 was evaluated with CCK8 kits (Additional file 1: Section：Concentration screening for PD-166866), with concentrations determined with reference to published studies.

### Wound healing assay

The wound healing assay was conducted according to a published protocol [28]. An inverted fluorescence microscope (CKX-41; Olympus Corp.) was used to image migration. GFP levels in the cells on the lower surface of the membrane were expressed as the mean ± standard error of the mean (SEM) from three separate experiments.

### Transwell assay

Cell invasion was examined using a Transwell assay as previously described [29]. HUVECs or CAFs were seeded in 500 μL serum-free DMEM in the lower chamber of the 24-well Transwell apparatuses. HUVECs (6 × 10^3^/well) or CAFs (5 × 10^3^/well) were suspended in 200 μl 10% FBS medium and seeded in the upper chamber. After co-culturing for 24 h, the migrated cells were fixed in methanol and stained with 0.1% crystal violet for 15 min. The cells on the membrane were imaged under a light microscope (model BX53; Olympus Corp.) and counted with Image J software.

### Immunofluorescence staining

Immunofluorescence staining followed the method described in our previous study [30]. Staining was examined by epifluorescence microscopy (model BX60-32FB2-A03; Olympus Corp.), and different filters were used for capturing images using an Olympus DP50 camera.

### Enzyme-linked immunosorbent assay

Proteins in the culture supernatants or mouse/patient sera were measured using enzyme-linked immunorobent assay ( (ELISA)) kits, as per the manufacturer’s instructions. Absorbances (450 nm) were determined using a microplate reader (Synergy HT; Biotek Instruments) (Additional File 1; Table S1).

### Endothelial cell tube formation assay

Tube formation assays are useful for assessing the angiogenic capacity of cells [31]. In the present study we used the branch point method. In brief, CRC cells were grown in the upper chambers of 24-well Transwell apparatuses (pore size: 0.4 µm) and HUVEC cells (5 × 10^4^/well) were grown in the lower chambers. The plates were pre-coated with Matrigel (50 µL) and cultured at 37 °C in a 5% CO_2_ incubator. After 12 h, the HUVECs were incubated with 1 mM calcein-acetoxymethyl ester (calcein-AM) for 1 h at 37 °C and examined under a fluorescence microscope (model CKX-41; Olympus Corp.) (Additional File 1; Table S1).

### Establishment of a CRC cell/MSC co-culture unit

A non-contact MSC and CRC cell co-culture system was constructed using a co-culture Transwell system (pore size: 0.4 μm) (upper chamber, CRC cells; lower chamber, MSCs) in which the culture medium was diffusible but the cells could not permeate. The medium was replaced at 48-h intervals. After 5 days, the cells in the lower chamber were collected [32].

### Xenograft tumor model

All of the animal experiments were performed according to the “Guide for the Care and Use of Laboratory Animals” (NIH Publication No. 80-23, revised 1996; National Academies Press, Washington DC, USA) and approved by the ethics committee of the Jiangsu Province Hospital of Chinese Medicine (no. 2021-10-029). Twenty-four 4-week-old male BALB/c nude mice were obtained from the Beijing Institute of Biomedicine (Beijing, China) (Certificate No. SYXK2019-0010). RKO cells transfected with sh-*COX4I2*, oe-*COX4I2*, as well as NC cells (1 × 10^7^ cell/mouse) were injected subcutaneously into the right armpit region (*n* = 6 per group). The presence of tumors was visible after 7 days. Tumor diameters (maximum and minimum) were measured twice a week. The mice were euthanized under CO_2_ on day 35, and the sera and tumors were excised. Euthanasia was fully consistent with the recommendations of the Guidelines on Euthanasia of the American Veterinary Medical Association [33]. Tumor volumes were calculated as *V* = 1/2*ab*^2^, and tumor growth curves were drawn.

### Transcriptomic expression and survival analysis

The differential expression of *COX4I2* and *COX19* (cytochrome c oxidase assembly factor COX19)in CRC tissue was investigated using The Cancer Genome Atlas (TCGA)-Colon Adenocarcinoma (COAD) cohort and the GSE10950 [34] (https://www.ncbi.nlm.nih.gov/geo/query/acc.cgi?acc=GSE10950) and GSE37182 [35] (https://www.ncbi.nlm.nih.gov/geo/query/acc.cgi?acc=GSE37182) datasets. Expression levels were analyzed by T (Tumor) stage, N (Node) stage, and M (Metastasis) according to the TCGA-COAD data (https://portal.gdc.cancer.gov/projects/TCGA-COAD) [36]. To investigate the effects of COX4I2 and COX19 on patient outcomes, survival analyses, including overall survival (OS), disease-free survival (DFS), and progression-free interval (PFI) were conducted using log-rank tests on the TCGA-COAD data.

### Least absolute shrinkage and selection operator and COX regression

Raw RNA-sequencing (RNA-Seq) data and clinical information on COX-related genes were downloaded from the TCGA dataset (https://portal.gdc.cancer.gov/) in January 2020. Least absolute shrinkage and selection operator (LASSO) analysis was performed with the R package ‘glmnet’ (R Foundation for Statistical Computing, Vienna, Austria). Univariate and multivariate Cox regression was conducted to identify the prognostic risk of *COX19* and *COX4I2*. Forest plots were determined using the “forestplot” R package.

### *COX4I2*-correlated gene enrichment analysis

High and low *COX4I2* expression groups were defined by the median cutoff in the TCGA-COAD dataset. The “DESeq” R package [37] was used to identify *COX4I2*-associated genes. The “clusterProfiler” R package [38] was used for functional enrichment analysis.

Gene set enrichment analysis (GSEA) was performed using the Broad Institute GSEA software 3.0 [39]. The gene set “subset of Gene Ontology (GO)” was acquired from the Molecular Signatures Databases (http://www.gsea-msigdb.org/gsea/msigdb/index.jsp) and was used for GO enrichment analysis. The false discovery rate (FDR) < 0.1 was considered to be statistically significant.

### Single-cell analysis

We downloaded a single-cell RNA-Seq dataset named CRC-016-01-1A (https://ngdc.cncb.ac.cn/cancerscem/browse) from the Database of Single-cell Expression Map (CancerSCEM), which consists of one CRC patient’s cells. All samples were imported into Seurat V3 and visualized in Uniform Manifold Approximation and Projection (UMAP) after a standardized quality control process. Next, the “FindAllMarkers” function (min.pct = 0.25, logfc.threshold = 2, tes.use = “wilcox”) was set to find markers for all cell types, and the “AddModuleScore” function was conducted to visualize gene set enrichment results. The “Dotplot” function was used to plot dot plots, and the “Vlnplot” function was used to plot violin plots.

### Fibroblast analysis

The Tumor Immune Estimation Resource (TIMER) 2.0 web tool (http://timer.cistrome.org) was used to evaluate the relationship between COX4I2 and fibroblasts [40]. Spearman’s rank correlation coefficient was calculated for pairwise correlation comparisons and *P* < 0.05 was considered to be statistically significant.

### Statistical analysis

Data are expressed as the mean ± SEM. Comparisons between two groups and multiple groups were assessed by *t*-tests and one-way analysis of variance, respectively. All data were analyzed with SPSS version 29.0 software (SPSS IBM Corp., Armonk, NY, USA) and illustrated using GraphPad Prism 8.0 (GraphPad Software, Inc., San Diego, CA, USA). All experiments were carried out at least three times. Statistical significance was determined as: ***P < 0.001, **P < 0.01 and *P < 0.05.

## Results

### Identification of the research targets

**Fig. 1:**
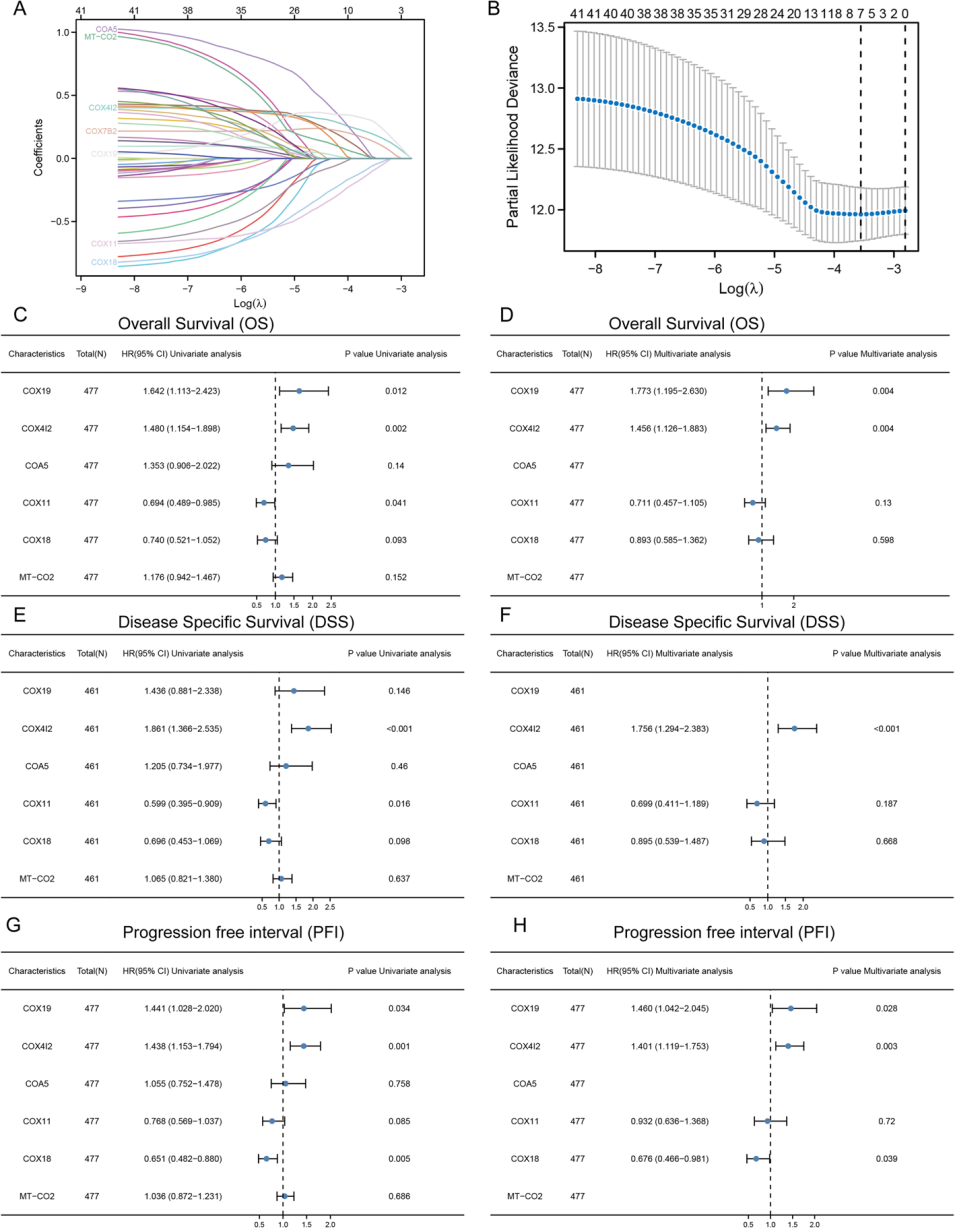
LASSO and Cox regression models. **a** LASSO Cox model fitting. Each curve represents a gene. The profiles of coefficients were plotted versus log(λ). Vertical lines indicate the positions of 7 genes with coefficients > 0, as determined by tenfold cross-validation (*n* = 480). **b** λ was determined from tenfold cross-validation. The* x*-axis represents log(λ); the *y*-axis represents binomial deviance. Optimal values calculated from minimum criteria and 1 SE of the criteria are indicated by the dotted vertical lines. **c**, **d** Univariate forest plot (**c**) and multivariate forest plot (**d**) showing association between *COX* expression and clinical features linked to OS in CRC (*n* = 477). **e**, **f** Univariate forest plot (**e**) and multivariate forest plot (**F**) showing the association between *COX* expression and clinical features related to DSS in CRC (*n* = 461). **g**, **h** Univariate forest plot (**g**) and multivariate forest plot (**h**) showing the association between *COX* expression and clinical features related to PFI in CRC (*n* = 477).* CI* Confidence interval,* HR *hazard ratio,* SE* standard error; for other abbreviations, see Abbreviation list

A total of 42 *COX*-related genes were used for LASSO regression to identify robust markers for follow-up studies. Cross-validation was performed in 10 rounds to prevent overfitting (Fig. 1a, b). The results of sequential univariate and multivariate Cox regression analyses showed that both *COX4I2* and *COX19* were associated with CRC prognosis, identifying these genes as research targets (Fig. 1c–h).

### Expression and survival analysis

**Fig. 2:**
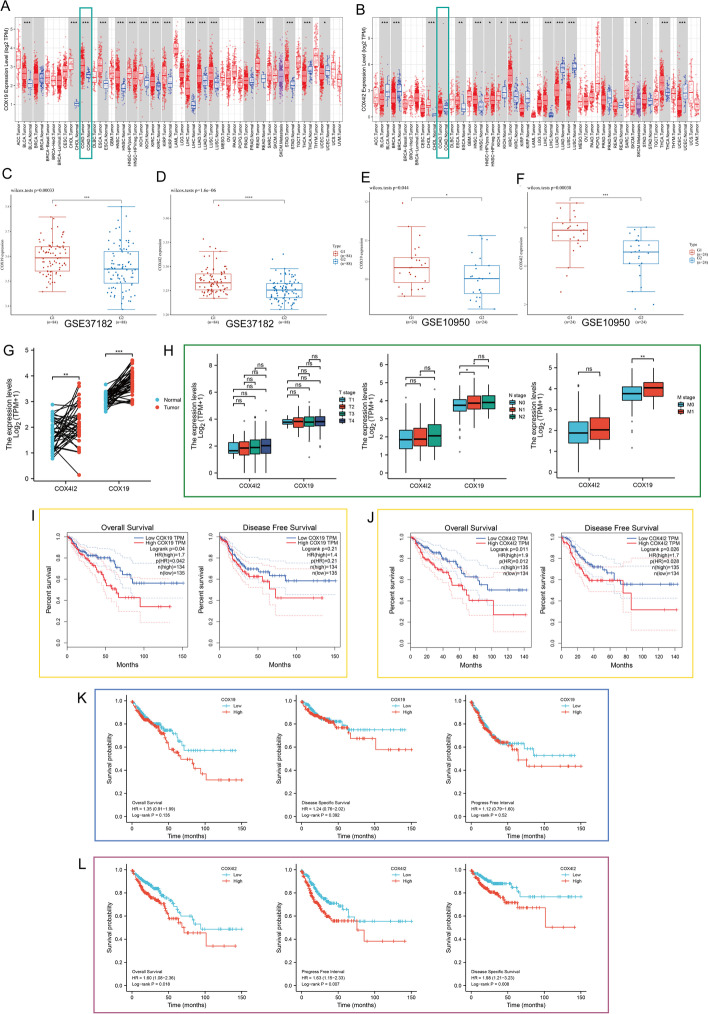
Expression of *COX19* and *COX4I2* in relation to clinical features of CRC. **a**, **b** *COX19* (**a**) and *COX4I2* (**B**) mRNA levels in various human tumor and normal tissues, from the TIMER database (*n* = 480). **c**, **d** Dataset GSE37182 (*n* = 172) from the GEO showing *COX19* (**c**) and *COX4I2* (**d**) mRNA levels in CRC. Wilcoxon test was performed. **e**, **f** Dataset GSE10950 (*n* = 48) from GEO showing *COX19* (**e**) and *COX4I2* (**f**) mRNA levels in CRC. Wilcoxon test was performed. **g** Expression of *COX19* and *COX4I2* in CRC, from TCGA-COAD (*n* = 41). **h** Association of *COX19* and *COX4I2* mRNA levels with the T (Tumor) (*n* = 477), N (Node) (*n* = 478), and M (Metastasis) (*n* = 411) stages. **i**, **j** Correlation of *COX19* (**i**) and *COX4I2* (**j**) levels with CRC patient OS and DFS, from the the GEPIA database (*n* = 269). **k**, **l** The overall survival, progression-free interval, and disease-specific survival from the TCGA-COAD data in relation to* COX19* (**k**) and* COX4I2* (**l**) expression (*n* = 477). Asterisks indicate significant difference at *P < 0.05, **P < 0.01, ***P < 0.001, ****P < 0.0001; *ns*, not significant.* GEO* Gene Expression Omnibus,* mRNA* messenger; for other Abbreviations, see Abbreviation list

The expression of *COX19* and *COX4I2* in various tumors was determined using the TIMER web tool. As shown in Fig. 2a, b, both of these genes were highly expressed in COAD. To further explore their expression in CRC tissues, data from the Gene Expression Omnibus (GEO) database were analyzed. As shown in Fig. 2c–f, the data extracted from GSE37182 and GSE10950 indicated that there was a significant difference between *COX19* and *COX4I2* expression in tumor tissues and controls (*P* < 0.05). The expression of the genes was further analyzed through TCGA, and the results showed that expression levels of both *COX19* and *COX4I2* were increased in COAD tissues compared with controls (Fig. 2g) (*P* < 0.01).

The association between *COX19* and *COX4I2* levels and clinicopathological characteristics is presented in Fig. 2h (factors including T/N/M stage). The mRNA expressions of *COX19* correlate with the N1, N2, and M1 stage, while *COX4I2* expression correlates with the N2 stage (*P* < 0.05).

Results from the Gene Expression Profiling Interactive Analysis (GEPIA) database showed both lower overall survival (OS; log-rank test, *P* = 0.011) and disease-free survival (DFS; log-rank test, *P* = 0.026) in the high *COX4I2* expression group, while high* COX19* levels were only associated with OS (log = rank test, *P* = 0.04) (Fig.2I, J). In addition, Kaplan–Meier (K–M) survival curves based on TCGA-COAD confirmed the relationship between elevated* COX4I2* and OS, DFS, and progression-free interval (PFI) (Fig. 2k, l).

### Functional enrichment analysis

**Fig. 3:**
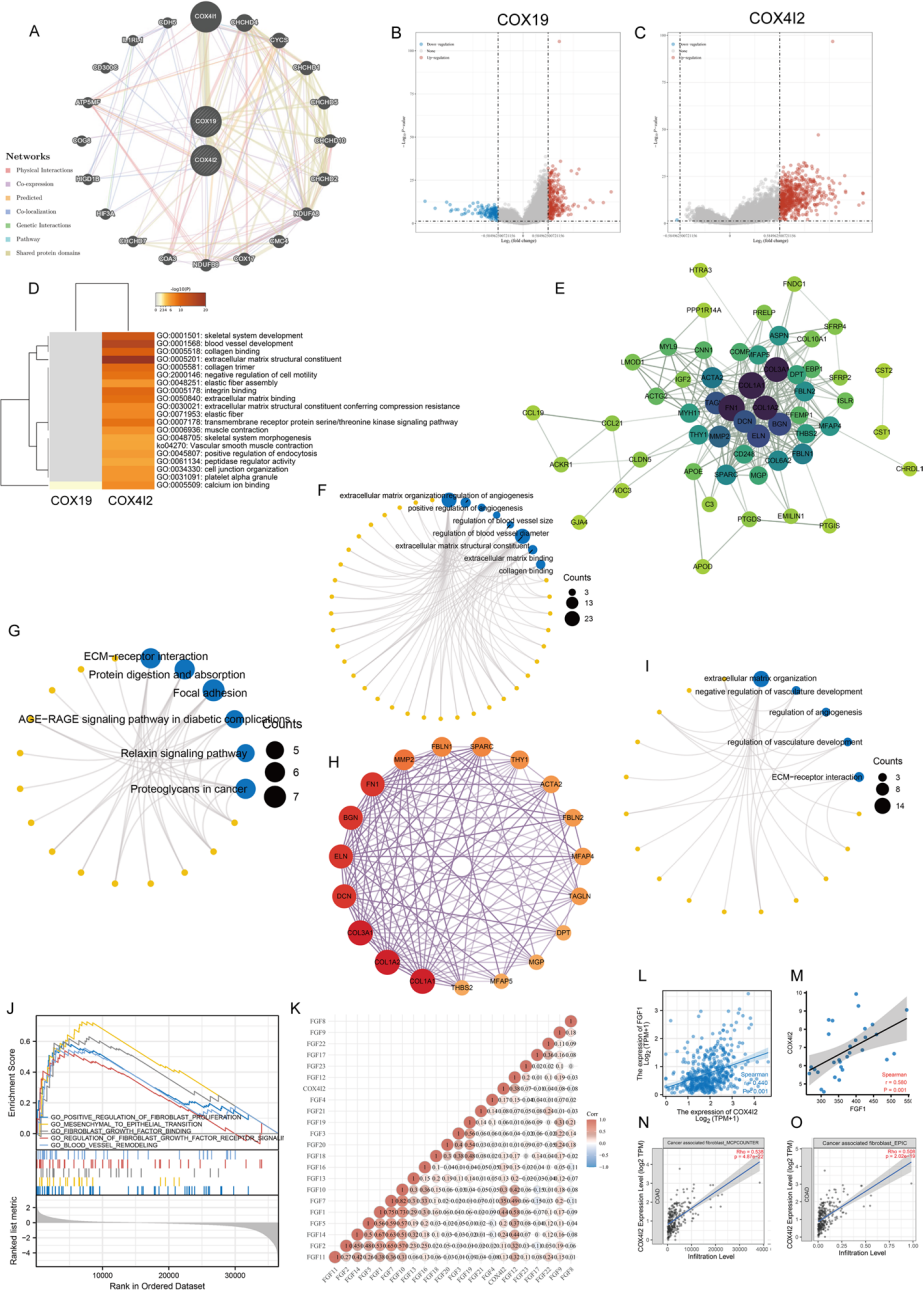
PPI network and enrichment analysis. **a** PPI constructed by GeneMANIA; *COX19* and *COX4I2* are at the core of this network. **b**, **c** Volcano map of differentially expressed genes after altered *COX19* (**b**) and *COX4I2* (**c**) levels. Red indicates upregulation, blue indicates downregulation. Data on the abscissa are differences in gene expression (log2 fold change); data on the ordinate represent the significance of these differences (− log10* P*adj). **d** GO and KEGG enrichment analysis of *COX19* and *COX4I2*. Each row represents 1 cluster; colors indicate significance, and gray color indicates non-significance. Heatmap showing the top 20 clusters correlated with *COX19* and *COX4I2*. **e** Network of *COX4I2* and genes significantly correlated with its expression. Darker colors and larger sizes indicate higher degrees of correlation. **f** The significantly enriched terms in GO analysis. **g** The significantly enriched terms in KEGG analysis. **h** The hub module with the highest scores analyzed by MCODE. **i** GO enrichment analysis of the module. **j** GSEA of *COX4I2*. **k** Correlation coefficient matrix of *COX4I2* and proteins of the FGF gene family. **l** Correlation between *COX4I2* and FGF1 expression. **m** Analysis of the correlation between the serum COX4I2 and serum FGF1 in the patients (*n* = 30) based on the comparison between the two groups. **n**, **o** Correlation of* COX4I2* levels with CAFs, from TIMER using the algorithms MCPCOUNTER (**n**) and EPIC (**o**).* MCODE* Molecular Complex Detection; for other Abbreviations, see Abbreviation list

A functional network based on functional predictions for *COX19* and *COX4I2* was constructed with GeneMANIA (Fig. 3a). The differentially expressed genes (DEGs) are shown in Fig. 3b, c. Enrichment analysis of the DEGs using METASCAPE suggested that *COX4I2* may be more involved in fibrosis-related pathways, such as elastic fiber, extracellular matrix, integrin, and collagen, compared to *COX19.* Consequently, *COX4I2* was selected for further study. The protein–protein interaction (PPI) network for *COX4I2* is shown in Fig. 3e. GO and KEGG analyses indicated the involvement of *COX4I2* in angiogenesis and stromal remodeling (Fig. 3f, g). The key subnetwork (Fig. 3h) obtained by the molecular complex detection (MCODE) plug-in showed a stronge association with angiogenesis (Fig. 3i). GSEA was then applied to further identify the possible mechanisms involved, and we observed that FGF may be closely associated with *COX4I2* (Fig. 3j). Finally, we calculated the Pearson correlation coefficient between *COX4I2* and all FGFs (Fig. 3k) at the transcriptomic level based on the TCGA-COAD data (Fig. 3l), from which we observed a significant correlation between *FGF1* and *COX4I2*, with the results from the CRC patients’ sera supporting this (Fig. 3m). Finally, we found a high positive correlation between *COX4I2* level and fibroblast abundance by MCPCOUNTER (*R* = 0.530, *P* = 4.87e−22; Fig. 3n) and EPIC methods (*R* = 0.508, *P* = 2.2e−19; Fig. 3o). These results implied that *CXO4I2* was closely associated with tumor fibrosis in CRC.

### Single-cell analysis

**Fig. 4:**
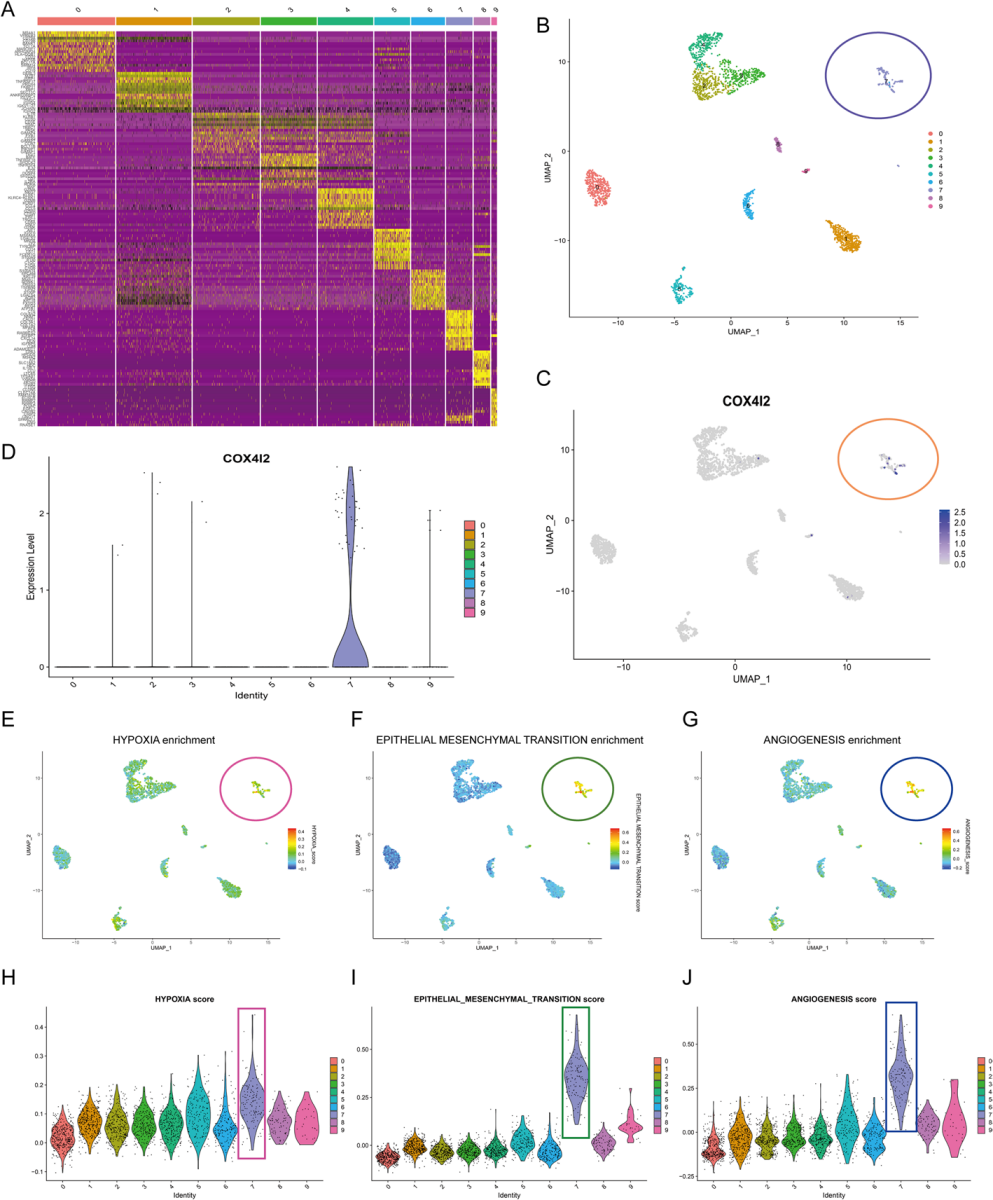
Single-cell analysis of *COX4I2* based on single-cell RNA sequencing dataset CRC-016-01-1A. **a** Cellular components. **b** UMAP plots showing different CRC cell types after quality control, reduction of dimensionality, and clustering. **c** UMAP plots showing expression of *COX4I2* clusters. **d** Violin plots of CRC cell cluster markers and *COX4I2* in various cell types. Expression was measured as log 2 (TP10K + 1). **e**–**g** Functional enrichment analysis. **h**–**j** Enrichment scores of genes from the Hallmark hypoxia gene set for each cell, from the gene set variation analysis: **h** HALLMARK_HYPOXIA,** i** EPITHELIAL_MESENCHYMAL_TRANSITION, **j** ANGIOGENESIS. For Abbreviations, see Abbreviation list

Single-cell profiling enables a better understanding of the composition of the CRC microenvironment. We investigated the association between *COX4I2* and FGFs using single-cell datasets. We identified additional genes that strongly and specifically marked each major cell population, as demonstrated in Fig. 4a. Figure 4b shows the cell markers of each cell population using UMAP. As seen in Fig. 4c, d, we observed, using UMAP and violin plots with unique markers for each cell type, that *COX4I2* was mainly expressed in cluster 7. As shown in Fig. 4e–g, functional enrichment analysis suggested that cells expressing *COX4I2* tended to express genes related to “HYPOXIA,” “EPITHELIAL-INTERSTITIAL-TRANSFORMATION,” and “ANGIOGENESIS”. The violin plot (Fig. 4h–j) shows that scores of “HYPOXIA,” “EPITHELIAL-INTERSTITIAL-TRANSFORMATION,” and “ANGIOGENESIS” were significantly higher in cluster 7 than in other clusters. Additional file 1: Fig.S1 also confirms an association between *COX4I2* and hypoxia-related gene expression and may contribute to an understanding of the potential mechanisms of *COX4I2*.

### *COX4I2* promotes malignant CRC phenotypes

**Fig. 5:**
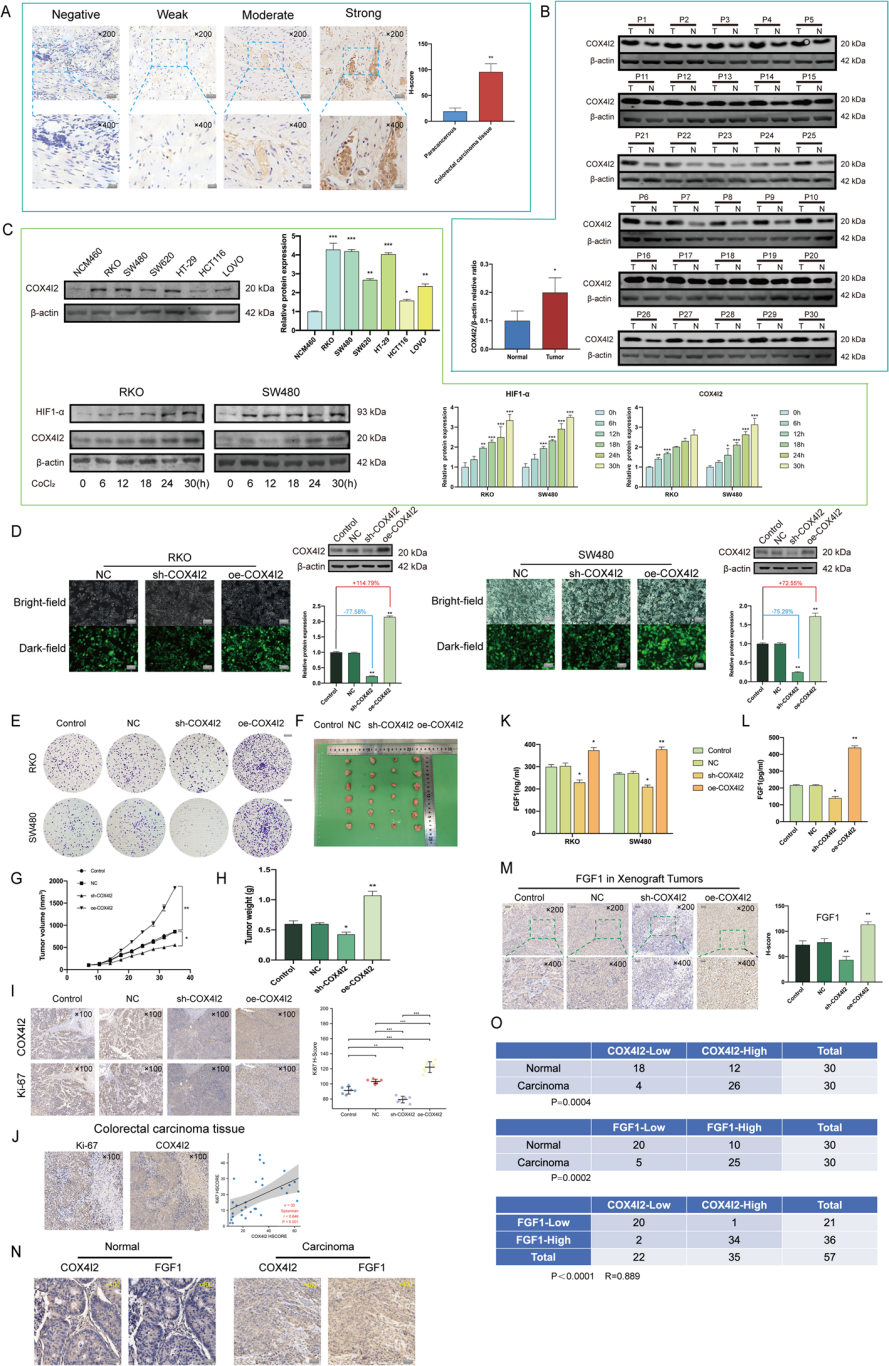
COX4I2 protein overexpression promotes CRC malignancy. **a** Analysis of IHC results and COX4I2 levels in CRC and control tissues (*n* = 30). **b** COX4I2 levels in tumor (*T*) and paired normal tissues (*N*) shown by western blotting (*n* = 30). **c** COX4I2 protein levels in normal human colonic epithelial cells and CRC cells lines. **d** Transfection efficiencies (%) shown by GFP expression and western blotting. (magnification: ×400, scale bars: 20 μm). **e** Clone formation in CRC cells transfected with the NC, sh-COX4I2, and oe-COX4I2 constructs (magnification: ×1, scale bars: 500 μm). **f** Mouse xenograft tumors (*n* = 6 mice per group). **g** Volumes of xenograft tumors measured twice a week. **h** Weights of xenograft tumors at completion of the study. **i**, **j** Correlation between IHC staining intensity for COX4I2 and Ki-67 in xenograft tumors (*n* = 24) and in CRC tissues (*n* = 30). Measures were taken from different samples. (magnification: ×100, scale bars: 100 μm). **k** FGF1 protein levels in culture supernatants of CRC cells 48 h after transfection, measured by ELISA. **l** FGF1 levels in mouse sera, measured by ELISA. Measurements were taken from distinct samples (*n* = 6). **m** IHC staining of FGF1 proteins in mouse xenograft tumor tissues (magnification: ×200, scale bars: 50 μm; magnification: ×400, scale bars: 20 μm). **n** COX4I2 and FGF1 protein levels in CRC and normal tissue (magnification: ×400, scale bars: 20 μm). **o** Correlation between COX4I2 and FGF1 in CRC. Statistical analyses were performed by the χ^2^ test (*R* Pearson correlation coefficient). Data are expressed as the mean ± standard error of the mean. Asterisks in all figure parts indicate significant difference at *P < 0.05, **P < 0.01, ***P < 0.001,. All IHC scores were repeated three times using a double-blind method. For the statistical analysis all experiments were repeated at least three times independently.* GFP* Green fluorescent protein,* oe-COX4I2* overexpressing plasmid,* RKO*,* SW480* cell lines,* sh-COX4I2* shRNA-COX4I2 plasmid); for other Abbreviations, see Abbreviation list

COX4I2 expression in CRC tissue was further explored using western blotting and IHC, which indicated high COX4I2 levels in both CRC cells and tissues. The mean (± SEM) H-SCOREs for COX4I2 expression in CRC and the paired control tissues were 95.53 ± 15.96 and 19.14 ± 6.65, respectively (Fig. 5a, b). COX4I2 levels were highest in the RKO and SW480 cell lines and were induced and activated by hypoxia in a time-dependent manner, consistent with HIF-1α (Fig. 5c). The efficiency of transfection was confirmed by GFP expression and western blotting (Fig. 5d). Silencing of *COX4I2* reduced clone formation (Fig. 5e). Moreover, stable *COX4I2* overexpression resulted in successful formation of xenograft tumors in mice (Fig. 5f–h). Ki-67 is a known prognosis-related factor for CRC, with strong expression linked to aggressive tumors and poor outcomes, and it is also a proliferation marker [41, 42]. We observed that CRC cases and tumors of nude mice with high *COX4I2* expression also exhibited strong Ki-67 staining signals (Fig. 5i, j). These results indicated that *COX4I2* may be correlated with Ki-67.

Based on the results of the enrichment analysis and observed correlation between *COX4I2* and *FGF1* in CRC patient sera, we speculated that *FGF1* may be involved in some of the cancer-associated behaviors regulated by *COX4I2*. Subsequently, we further verified the correlation of *COX4I2* with *FGF1* using culture supernatants (Fig. 5k), mouse sera (Fig. 5l), mouse tumors (Fig. 5m), and CRC tissues (Fig. 5n, o). COX4I2 expression was found to be strongly correlated with FGF1 expression in all cases.

### *COX4I2* overexpression promoted the epithelial–mesenchymal transition process

**Fig. 6:**
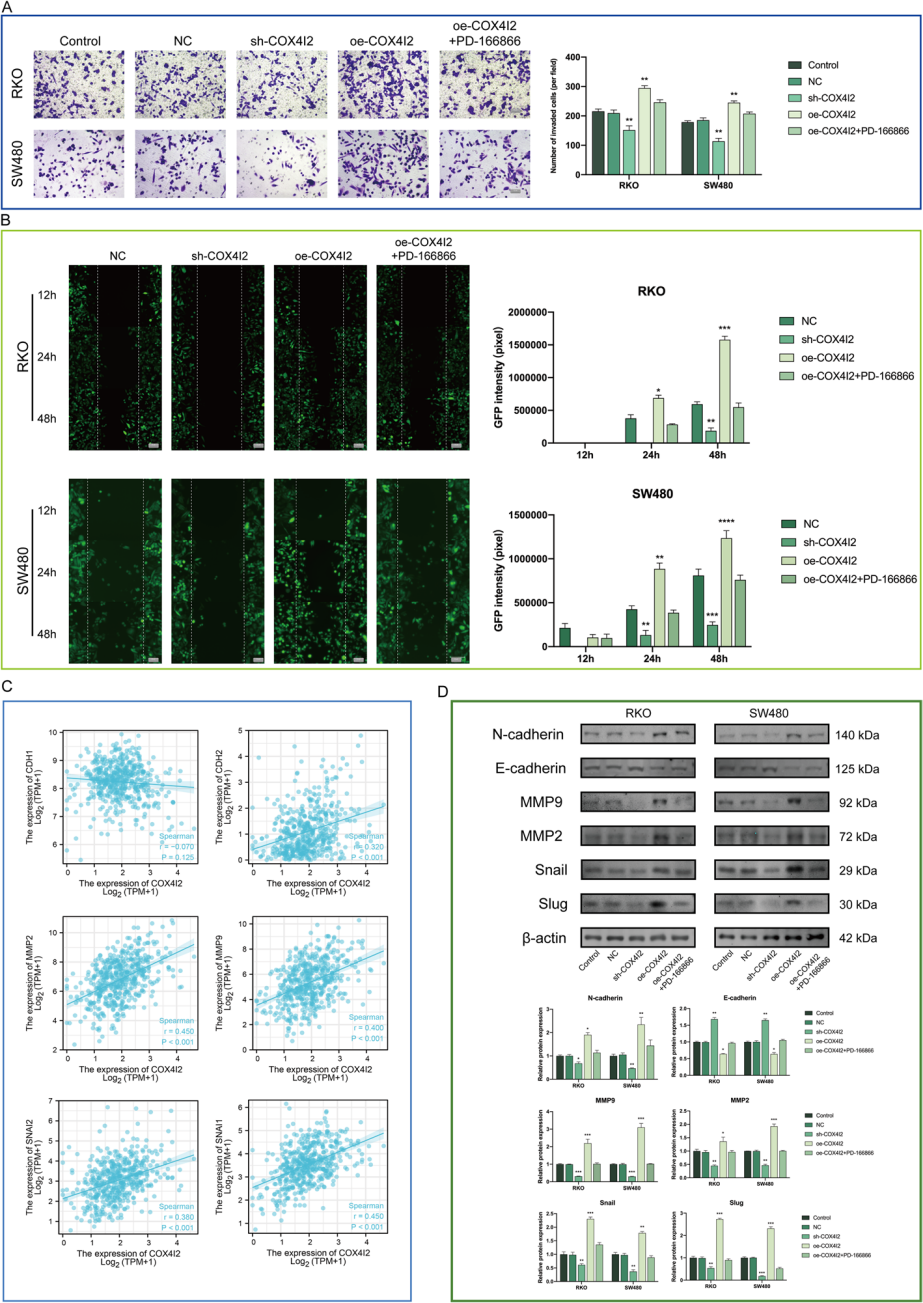
Overexpression of *COX4I2* promotes CRC cell invasion and metastasis by affecting the EMT. **a** Cell invasion (Transwell assay) (magnification: × 200, scale bars: 50 μm). **b** Cell migration (scratch assay) (magnification: × 200, scale bars: 50 μm). **c** Correlations between *COX4I2* and EMT-related genes, from TIMER. **d** EMT-related proteins shown by western blotting (*n* = 3 replicates). Data are expressed as mean ± SEM. Asterisks indicate significant difference at *P < 0.05, **P < 0.01, ***P < 0.001, ****P < 0.0001. All experiments were repeated at least three times, independently. For abbreviations, see Abbreviation List

GSEA and single-cell RNA-Seq analyses showed that *COX4I2* expression was closely correlated with the epithelial–mesenchymal transition (EMT) process. Increases in EMT-related phenotypes were found to be linked to *COX4I2* overexpression. This influence decreased with treatment with the integrin FGF1-specific inhibitor PD-166866 (Fig. 6a, b). Addition of FGF1 (10 ng/mL) rescued *COX4I2* knockdown CRC cells EMT phenotype (Additional file 1: Fig. S2). The cell viability assays showed that cell proliferation was not influenced at the chosen PD-166866 concentration (Additional file 1: Fig.S3; Additional file 1: Fig.S4). Correlation analysis (Fig. 6c) showed that *COX4I2* expression was positively associated with *CDH2* (*R* = 0.320, *P* < 0.001), *MMP2* (*R* = 0.450, *P* < 0.001), *MMP9* (*R* = 0.400, *P* < 0.001), *SNAI2* (*R* = 0.380, *P* < 0.001), and *SNAI1* (*R* = 0.450, *P* < 0.001) expression and negatively linked with *CDH1* expression (*R* =  − 0.070, *P* = 0.125), in agreement with the in vitro observations. These results were confirmed in vivo at the protein level (Fig. 6d).

### COX4I2 promotes angiogenesis and CAF activation

The TFacts tool (http://www.tfacts.org/) was employed to predict transcription factors that regulate COX4I2-related gene expression in CRC, using all genes that were found to be positively correlated with *COX4I2* in the COAD dataset of TCGA. This analysis showed that transcription factors involved in angiogenesis and hypoxia-related signaling pathways were significantly enriched, such as SMAD2/7 and NF-κB1. The enrichment analysis also suggested that these transcription factors are closely associated with angiogenesis and CRC (Additional file 1: Fig.S5).

**Fig. 7:**
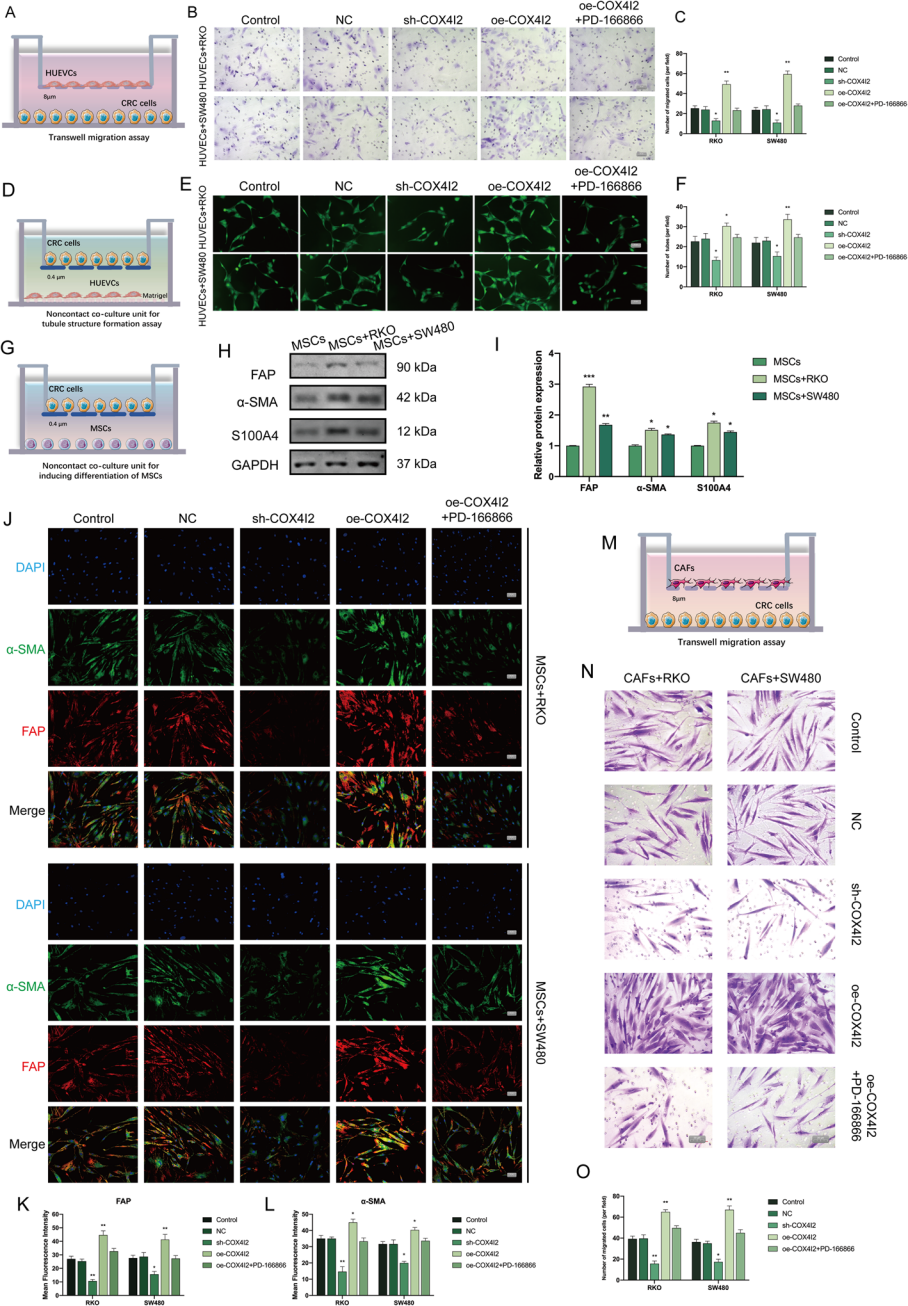
Overexpression of COX4I2 promotes angiogenesis and CAF activation in CRC. **a** The HUVEC migration assay. **b**, **c** HUVEC migration analysis (Transwell assay) (magnification: ×200, scale bars: 50 μm). **d** The tube formation assay. **e**, **f** HUVEC tube formation and graph showing tube formation indices in different groups (magnification: ×200, scale bars: 50 μm). **g** Non-contact co-culture of MSCs and CRC cells. Cells were used in a 1:1 ratio. **h**, **i** Western blot showing CAF-related markers after 5 days of co-culture (*n* = 3 replicates). **j**–**l** Immunofluorescence of MSCs co-cultured with CRC cells (control cells and CRC cells transfected with the NC, sh-COX4I2, and oe-*COX4I2* constructs) treated with or without 5 µM PD-166866 (magnification: ×400, scale bars: 20 μm). **m** The CAF migration assay. **n**, **o** Recruitment of CAFs by CRC cells (Transwell) (magnification: ×200, scale bars: 50 μm). Cell numbers were assessed using Image J. Data are expressed as the mean ± SEM. Asterisks indicate a significant difference at *P < 0.05, **P < 0.01, ***P < 0.001. All experiments were repeated at least three times, independently. For Abbreviations, see Abbreviation list

Considering these results, and also to verify the GSEA and single-cell RNA-Seq analyses of angiogenesis and fibroblast activation, we established several in vitro co-culture models. We first noted that overexpression of *COX4I2* in CRC promoted the migration of HUVECs (Fig. 7a–c) as well as their ability to form blood vessels (Fig. 7d–f). This malignant phenotype could be rescued by inhibitors of FGF1. CAFs, which are known for their properties in forming the tumor microenvironment, originate from circulating MSCs. The non-contact co-culture model (Fig. 7g) showed that *COX4I2* overexpression could promote the differentiation of MSCs to CAFs, which was mainly reflected by a significant increase in markers of CAF activation in the co-culture system (Fig. 7h–l). Fibroblast activation protein (FAP), fibroblast-specific protein-1 (S100A4), and α-smooth muscle actin (α-SMA) are all commonly used to label activated CAFs. As we have previously demonstrated, these observations also showed that pretreatment with an FGF1 inhibitor reduced MSC differentiation into CAFs in the co-culture unit. The invasion of cancer cells not only refers to the tumor cells themselves, but also encompasses the recruitment of endothelial cells and fibroblasts in the surrounding stromal microenvironment. Migration assays confirmed that *COX4I2* overexpression increased CAF recruitment by the cancer cells (Fig. 7m–o). Additional addition of FGF1 (10 ng/mL) rescued the phenotype after knockdown of COX4I2, including angiogenesis and fibroblast activation (Additional file 1: Fig.S6).

Together, these findings indicated that COX4I2 promotes CRC progression by stimulating angiogenesis and fibroblast activation.

## Discussion

Colorectal cancer is a highly invasive and heterogeneous disease [43]. There are several aspects to its heterogeneity. On the one hand, the disordered proliferation of the cancer cell leads to increased oxygen consumption and hypoxia in the local microenvironment, stimulating angiogenesis and the formation of abnormal tumor-associated blood vessels [44]. On the other hand, tumor-associated stromal cells are exposed to the same hypoxic microenvironment as the tumor parenchymal cells and contribute to tumor progression [45]. As one of the most striking features of the tumor microenvironment, hypoxia represents an adaptive mechanism for tumor development that drives tumor progression and adversely affects treatment outcome [46, 47]. There are currently no methods to quantify or determine the degree of tumor hypoxia. Therefore, the development of hypoxia-related biochemical markers could help improve our understanding of the biological mechanisms of CRC and the management of CRC therapy.

Mammals rely heavily on aerobic respiration for their energy needs, with most of the inhaled oxygen being used by COX [48] which, as the last enzyme in the respiratory chain, must cope with fluctuating oxygen concentrations [49]. Similar response mechanisms also exist for cancer cells, and these are closely linked to the malignant properties of cancer cells, of which one of the best known aspects is the ability of increased ROS production through the mitochondrial respiratory respiratory chain to strongly drive cancer cell invasion and metastasis [50, 51]. This activity indicates the likely involvement of respiratory chain complexes in remodeling the hypoxic tumor microenvironment. We therefore performed a LASSO-COX regression based on TCGA-COAD data and identified *COX4I2* as well as *COX19* in respiratory chain complexes as potential cancer risk factors. There is little documentation of these two genes in relation to tumors, and the topic is worthy of investigation. We then compared the potential biological functions of both genes by functional enrichment analysis, which indicated the likely involvement of* COX4I2* with multiple fibrosis-associated pathways, suggesting that this gene would be a noval target of further investigation. The induction of *COX4I2* expression under hypoxic conditions has been reported in numerous cell lines, and it has been proposed that the COX4 isoform is specifically linked to stimulating mitochondrial respiration in response to reduced oxygen. Our findings showed that *COX4I2* is a significant risk factor in CRC, and we speculate that it may play a similar role in oxygen adaptation in CRC.

The analysis of survival data showed that elevated *COX4I2* expression was related to unfavorable CRC prognosis and that *COX4I2* levels were significantly elevated in CRC tissues at both the transcriptomic and proteomic levels. Ki67 is involved in early rRNA synthesis, has been linked to a variety of signaling pathways, and is induced by hypoxia [52, 53]. We used Ki67 as a positive control, although Ki67 expression does not show a homogeneous pattern in all tissues (related to the spatial heterogeneity and temporal variability of hypoxia in tumors), We found that *COX4I2* expression was highly consistent with Ki67 expression and that *COX4I2* overexpression significantly stimulated the proliferative capacity of CRC cells. We hypothesize that *COX4I2* may be a downstream factor of hypoxia-triggered signaling cascades involved in the onset of cancer proliferation.

Enrichment analysis suggested that *COX4I2* may be associated with the activation of FGFs, and evidence at the single-cell level implied a close link between *COX4I2* and hypoxia, EMT, and angiogenesis. Calculation of the correlations between *COX4I2* and all FGF family members based on TCGA-COAD revealed that the closest link may exist between *COX4I2* and *FGF1*. *FGF1* has oncogenic activity and has been found to modulate numerous cellular processes, including proliferation, invasion, and survival [54]. *FGF1* levels are elevated in a variety of tumors and the gene is implicated in malignant progression [55]. In tumors, *FGF1* may be derived from both tumor and surrounding stromal cells and may have both paracrine or autocrine activity [56]. Secreted FGF1 binds and activates specific FGF receptors (FGFRs) which have tyrosine kinase activity, with intrinsic tyrosine kinase activity on the cell surface initiating signal transduction cascades with various effects, including the promotion of the EMT [57]. It has been shown that *FGF1* can promote angiogenesis and fibroblast differentiation [58, 59]. Activated fibroblasts can further secrete FGF1, contributing to a vicious cycle of malignant phenotypes [60]. Evidence from clinical samples as well as in vivo experiments both suggested that *COX4I2* acts as a powerful FGF simulator for FGF1, a fact that we also consider being a likely reason for *COX4I2*’s link to poor prognosis.

It has been well established that *FGF1* promotes the metastasis of various tumors through the EMT [61]. The role of the EMT in neovascularization is more complex [62, 63]. Because the process of tumor metastasis is a continuation of the cascade response, it can be envisaged that the end of one cascade will feed into the beginning of the next [6]. For example, SNAIL1-mediated EMT may contribute to neoangiogenesis in the case of metastases that rely on an angiogenic pattern rather than the EMT [64]. In our study, the Transwell and wound-healing assays confirmed the ability of *COX4I2* to activate the EMT via the FGF1 pathway, a conclusion reinforced by calculations based on the TCGA-COAD data and in vitro experiments.

FGF1 is a non-classically released growth factor which is essential for vascular endothelial cell proliferation and is an even more potent angiogenic factor than VEGF or platelet-derived growth factors [65, 66]. We clarified that *COX4I2* can promote angiogenesis by regulating the level of FGF1. CAFs originate from circulating MSCs [67] and are involved in the formation of tumor-associated stroma [68]. The differentiation of tumor-infiltrating MSCs to CAFs can be facilitated by FGF1. CAFs can further secrete FGF1 to actively participate in tumor-stromal crosstalk and further drive tumor progression [69]. The results of different algorithms in our study all indicated a significant relationship between *COX4I2* levels and the degree of fibroblast infiltration. Also, in the co-culture system we established, we obtained direct evidence that elevated *COX4I2* expression enhanced the recruitment and activation of fibroblasts.

## Conclusion

This study represents a preliminary exploration of the possible role of *COX4I2* in CRC, with the results revealing the complexity of its underlying mechanisms. Although there are still many questions about how *COX4I2* affects FGF1, we present evidence that *FGF1* is an important molecule in the signaling network in which *COX4I2* is involved. In addition, how tumor cells adapt to hypoxia through COX in the tumor microenvironment is an interesting and significant topic, and our subsequent work will focus on these two aspects to try to elucidate the exact pro-cancer mechanism of *COX4I2*.

## Supplementary Information

**Additional file 1. Table S1**.** Figure S1**.** Figure S2**.** Figure S3**.** Figure S4**.** Figure S5**.** Figure S6**.

## Data Availability

Jie-pin Li can be contacted (zjgzy027@njucm.edu.cn) regarding the availability of data and materials.

## Abbreviations

CAFs: Cancer-related fibroblasts
COAD: Colon adenocarcinoma
COX: Cytochrome *c* oxidase
*COX19*: Gene encoding cytochrome* c* oxidase assembly factor COX19
COX4I2: Cytochrome *c* oxidase subunit 4 isoform 2 encoded by the* COX4I2* gene
CRC: Colorectal cancer
DFS: Disease-free survival
DMEM: Dulbecco’s Modified Eagle Medium
ELISA: Enzyme-linked immunosorbent assay
EMT: Epithelial–mesenchymal transition
FBS: Fetal bovine serum
FDR: False discovery rate
FGF1: Fibroblast growth factor 1
HIF: Hypoxia-inducible factor
HUVECs: Human umbilical vein endothelial cells
GEPIA: Gene expression profiling interactive analysis
GSEA: Gene set enrichment analysis
GO: Gene Ontology
IHC: Immunohistochemistry
KEGG: Kyoto Encyclopedia of Genes and Genomes
LASSO: Least absolute shrinkage and selection operator
MAPK: Mitogen-activated protein kinase
MMPs: Matrix metalloproteinases
MSCs: Mesenchymal stromal cells
NC: Non-targeting plasmid
NF-κB: Nuclear factor kappa-B
OS: Overall survival
PDGFs: Platelet-derived growth factors
PFI: Progression-free interval
PPI: Protein–protein interaction
ROS: Reactive oxygen species
shRNA: Short hairpin interfering RNA
STR: Short tandem repeat
TCGA: The Cancer Genome Atlas
TIMER: Tumor Immune Estimation Resource
UMAP: Uniform manifold approximation and projection
VEGF: Vascular endothelial growth factor

## Declarations

### Adherence to ARRIVE guidelines

Our study complies with the ARRIVE guidelines (https://arriveguidelines.org).
