# Supplementary material for: Identification of COX4I2 as a hypoxia-associated gene acting through FGF1 to promote EMT and angiogenesis in CRC
Source: Cell Mol Biol Lett. 2022 Sep 5;27:76. doi: 10.1186/s11658-022-00380-2 (PMC9446847; doi:10.1186/s11658-022-00380-2)
Supplement: Supplementary file 1 — Additional file 1. Table S1. Figure S1. Figure S2. Figure S3. Figure S4. Figure S5. Figure S6. [file 11658_2022_380_MOESM1_ESM.pdf]

Additional file

Table S1

Antibodies and Reagents	Manufacturer, Country, Batch number	Concentration
Roswell Park Memorial Institute 1640 (RPMI-1640)	Gibco, USA, Lot: 8121248	-
Dulbecco's modified Eagle's medium (DMEM)	Gibco, USA, Lot: 8121032	-
Fetal bovine serum (FBS)	Gibco, USA, Lot: 42F1376K	-
Penicillin/streptomycin	Gibco, USA, Lot: 2321125	
GMyc-PCR Mycoplasma Test Kit	YEASEN, China, Lot: G5110070	
Plasmid vectors for COX4I2	GeneChem, China, Lot: GIEE0304606	
Puromycin	Beyotime Biotechnology, China, Lot: 041321210517	1.5 µg/mL
Lipofectamine 3000	Invitrogen, USA, Lot: 2395298	-
Opti-MEM	Gibco, USA, Lot: 2276923	-
COX4I2 Antibody	Invitrogen, USA, Lot: 3113BA09	WB: 1:1000<br>IHC: 1:200
Fibroblast Growth Factor 1 (FGF1) ELISA Kit	Colorful Gene Biological Technology, China, Lot: GR2021-09	-
Anti-β-actin Antibody	Invitrogen, USA, Lot: RI2265993	WB: 1:5000
Anti-E-cadherin Antibody	Cell Signaling Technology, USA. Lot:6	WB: 1:1000
HIF1A Polyclonal Antibody	Thermo Fisher Scientific, USA, Lot: VL3162393	WB: 1:1000
Anti-N-cadherin Antibody	Cell Signaling Technology, USA.	WB: 1:1000

	Lot:12	
Anti-MMP2 Antibody	Cell Signaling Technology, USA. Lot:3	WB: 1:1000
Anti-MMP9 Antibody	Cell Signaling Technology, USA. Lot:5	WB: 1:1000
Anti-Snail Antibody	Invitrogen, USA, Lot: PJ19211801	WB: 1:1000
Anti-Slug Antibody	Invitrogen, USA, Lot: QI2093091	WB: 1:1000
Anti-FAP Antibody	Invitrogen, USA, Lot:30E12A05	WB: 1:1000
Anti-α-SMA Antibody	Cell Signaling Technology, USA. Lot:3	WB: 1:1000
Anti-S100A4 Antibody	Invitrogen, USA, Lot: MAB02754	WB: 1:1000
Anti-Ki-67 Antibody	AiFang Biological, China, Lot: AF20068	IHC: 1:200
Anti-FGF1 Antibody	AiFang Biological, China, Lot: AF02204	IHC: 1:200
PD-166866	Selleck Chemicals, USA, Lot: S8493	-
0.2% TritonX-100	Proteintech, China, Lot: 61202011	-
Cobalt (II) chloride, anhydrous, 97%	Thermo Fisher Scientific, USA, Lot: B22031	300 μmol/L
Human FGF1 ELISA Kit.	Aifang Biotechnology, China, Lot: J12034	
Matrigel	BD Biosciences, USA, # 07898	
Recombinant Human FGF-basic (154 a.a.)	Peprotech, USA. Lot: 0820AFC05	10ng/ml
Nuclei were stained using 4',6-diamidino-2-phenylindole (DAPI)	Beyotime Biotechnology, China, Lot: 091620210520	-
FAP Antibody for	AiFang Biological, China, Lot:	IF: 1:500

Immunofluorescence	AF301279	
a-SMA Antibody for Immunofluorescence	AiFang Biological, China, Lot: AF300331	IF: 1:500
Calcein-acetoxymethyl ester (calcein-AM)	Solarbio LIFE SCIENCES, China, Lot: 20210810	-
Alexa Fluor 488 AffiniPure goat anti-mouse IgG (H+L)	FcMACS, China, Lot: 136908	1:2000
Goat anti-mouse IgG (H+L) CoraLite594	Proteintech, China, Lot: 20000154	1:1000
Goat anti-rabbit IgG (H+L) R-PE conjugate	Proteintech, China, Lot: 20000129	1:1000

Immunohistochemical staining (IHC)

Sections were blocked with blocking solution and incubated with anti- COX4I2, anti-Ki-67, and anti-FGF1 antibodies. The extent and intensity of staining were assessed by two independent investigators. Staining intensities were graded as 0 (none), 1 (weak), 2 (moderate), and 3 (strong). Staining extent was graded as 0 (no positively-stained cells), 1 (less than 10%), 2 (10-50%), and 3 (over 50%). The histochemistry score (H-SCORE), representing both the proportion of stained cells and the degree of staining, was determined as: H-SCORE= $\sum(PI \times I)$ = (percentage of cells with weak intensity × 1) + (percentage of cells with moderate intensity × 2) + (percentage of cells with strong intensity × 3), where PI represents the percentage of positive cells to the total number of cells in a particular field and I represent the intensity of staining. The H-SCORE ranges between 0 to 300, with higher scores indicating stronger staining.

Western blotting

Cells were lysed in RIPA buffer and protein concentrations measured by the Bradford assay. Samples of 20 μg each were separated on 10% or 8% SDS-PAGE. Proteins were transferred to polyvinylidene fluoride (PVDF) membranes and blocked with 5% bovine serum albumin. The blots were then probed with the relevant primary antibodies at 4°C overnight. After washing three times in Tris-buffered saline containing 0.05% Tween-20, the blots were incubated with the corresponding secondary antibodies and an Electrochemiluminescence (ECL) detection kit used to measure densities. The β-actin protein was used as a reference.

COX4I2 RNAi and overexpression preparation

Gene information:

Gene symbol	GenBank_ID
COX4I2	NM_032609

Target information:

ID	Target Sequence information：
COX4I2-RNAi(105769-1)	gaTTCGCAGCTCTGGTGATTT
COX4I2-RNAi(105770-1)	gcTCCAGTTCAATGAGACCTT
COX4I2-RNAi(105771-1)	gcCCTTCTGCACAGAACTCAA

shRNA Sequence：

ID	5’	stem	loop	stem	3’
COX4I2-RNAi(105769-1)-a	Ccgg	gaTTCGCAGCTCTGGTGATTT	CTCGAG	AAATCACCAGAGCTGCGAATC	TTTTTg
COX4I2-RNAi(105769-1)-b	aattcaaaaa	gaTTCGCAGCTCTGGTGATTT	CTCGAG	AAATCACCAGAGCTGCGAATC	
COX4I2-RNAi(105770-1)-a	Ccgg	gcTCCAGTTCAATGAGACCTT	CTCGAG	AAGGTCTCATTGAACTGGAGC	TTTTTg
COX4I2-RNAi(105770-1)-b	aattcaaaaa	gcTCCAGTTCAATGAGACCTT	CTCGAG	AAGGTCTCATTGAACTGGAGC	
COX4I2-RNAi(105771-1)-a	Ccgg	gcCCTTCTGCACAGAACTCAA	CTCGAG	TTGAGTTCTGTGCAGAAGGGC	TTTTTg
COX4I2-RNAi(105771-1)-b	aattcaaaaa	gcCCTTCTGCACAGAACTCAA	CTCGAG	TTGAGTTCTGTGCAGAAGGGC	

Plasmid name： GV493

Element’s order： hU6-MCS-CBh-gcGFP-IRES-puromycin

Negative control number： CON313

Negative control insert sequence： TTCTCCGAACGTGTCACGT

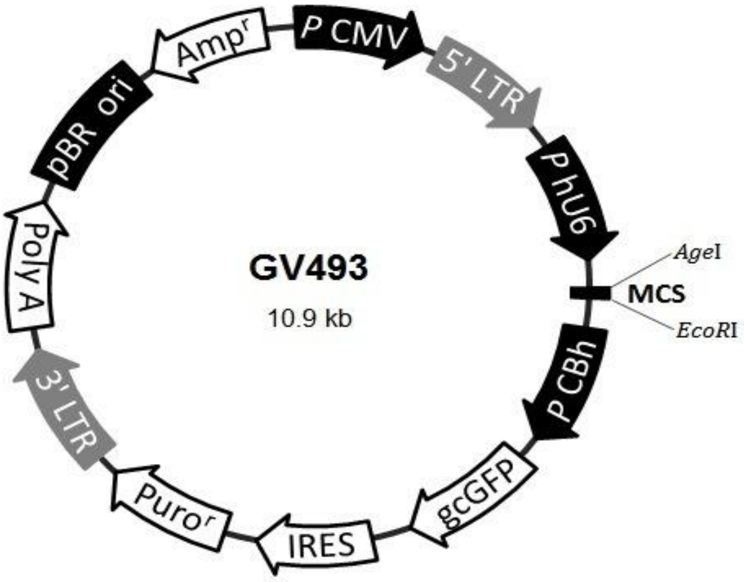

Overexpression Plasmid information：

Plasmid name： GV657

Element's order: CMV enhancer-MCS-3flag-polyA-EF1A-zsGreen-sv40-puromycin

The cloning site: BamHI / KpnI

Control number： CON468

The positive clones sequencing results were analyzed

The comparison results were shown as follows：

CGAGCGGCCGCCACTGTGCTGGATATCTGCAGAATTCCACCACACTGGACTAGTGGATCCCGC
CACCATGCTCCCCAGAGCTGCCTGGAGCTTGGTGCTGAGGAAAGGTGGAGGTGGAAGACGAG
GGATGCACAGCTCAGAAGGCACCACCCGTGGTGGGGGGAAGATGTCCCCCTACACCAACTGCT
ATGCCCAGCGCTACTACCCCATGCCAGAAGAGCCCTTCTGCACAGAACTCAACGCTGAGGAGC
AGGCCCTGAAGGAGAAGGAGAAGGGAAGCTGGACCCAGCTGACCCACGCCGAAAAGGTGGC
CTTGTACCGGCTCCAGTTCAATGAGACCTTTGCGGAGATGAACCGTCGCTCCAATGAGTGGAAG
ACAGTGATGGGTTGTGTCTTCTTCTTCATTGGATTCGCAGCTCTGGTGATTTGGTGGCAGCGGGT
CTACGTATTTCCTCCAAAGCCGATCACCTTGACGGACGAGCGGAAAGCCCAGCAGCTGCAGCG
CATGCTGGACATGAAGGTGAATCCTGTGCAGGGCCTGGCCTCCCGCTGGGACTATGAGAAGAA
GCAGTGGAAGAAGTCGGTACCAAGCTTAAGTGACTACAAGGATGACGATGACAAGGATTACAA
AGACGACGATGATAAGGACTATAAGGATGATGACGACAAATCTAGATAGTTAATTAAACCGGTA
ATAAAATATCTTTATTTTCATTACATCTGTGTGTTGGTTTTTTGTGTGAATCGATAGTACTAACATA
CGCTCTCCATCAAAACAAAACGAAACAAAACAAACTAGCAAAATAGGCTGTCCCCAGTGCAAG
TGCAGGTG

ELISA

ELISA kits were used to measure proteins in culture supernatants and sera of patients and mice. Culture supernatants were centrifuged for 20 min at 2000 rpm in sterile tubes. Blood samples were centrifuged (2500 rpm for 15 min) and the sera frozen at -80 °C. ELISAs were used to measure COX4I2/FGF1 concentrations. Ninety-six-well plates were coated with conditioned medium at 4 °C overnight, washed three times with PBS-Tween, and blocked with BSA at 37 °C for 2 h. After rinsing, anti-FGF1 antibody was incubated at 4 °C overnight, the plates were again washed, and incubated with HRP-conjugated secondary antibody for 2 h at room temperature and 100 µL of freshly prepared solution containing peroxidase was added. The enzyme was blocked with 100 µl H_2SO_4·2 N and absorbances read at 450 nm with a microplate reader. The concentrations of COX4I2/FGF1 were calculated from standard curves.

Wound healing assay

SW480/GFP and RKO/GFP cells were used in a wound-healing assay to measure migration ability. The CRC cells were seeded in 6-well plates at a density of 4×10^5 cells per well and grown to confluence for 24 h in serum-free medium. After removal of the medium, the cell layer was scratched with a 10 µL pipette tip. Wound-healing was examined under an an inverted fluorescence microscope (Olympus CKX-41, Japan) (×200 magnification) after 12, 24, and 48 h.

Colony formation assays

Five hundred cells were seeded in a 6-well plate and cultured for about 14 days. Colonies (>5 cells per colony) were stained with 0.5% crystal violet at room temperature (20-25°C) for 10 min and counted using a stereomicroscope.

Immunofluorescence staining

Cells were seeded on coverslips, fixed in chilled methanol for 10 min, and permeabilized with 0.1% Triton X-100 for 10 min at room temperature. After blocking (10% BSA in PBS), the coverslips were incubated with primary antibody overnight (4 °C), followed by incubation with the corresponding secondary antibodies. Nuclei were counterstained with DAPI (3 min in the dark) and the slides were washed in PBS and sealed with a solution containing a fluorescence quencher. Slides were examined and imaged under a fluorescence microscope.

Fig. S1. Correlations between the expression of COX4I2 and hypoxia-related genes

(A) Hypoxia-inducible aryl hydrocarbon receptor nuclear translocator (ARNT). This gene is also a co-factor for transcriptional regulation by hypoxia-inducible factor 1 (HIF-1β).
(B) EPAS1 (Endothelial PAS Domain Protein 1 also called Hypoxia-Inducible Factor 2 Alpha,

HIF-2-Alpha). This gene encodes a transcription factor that induces expression of oxygen-related genes.

(C) Hypoxia Inducible Factor 1 Subunit Alpha (HIF-A). This gene encodes the alpha subunit of HIF-1, which regulates oxygen homeostasis by promoting the transcription of various genes.

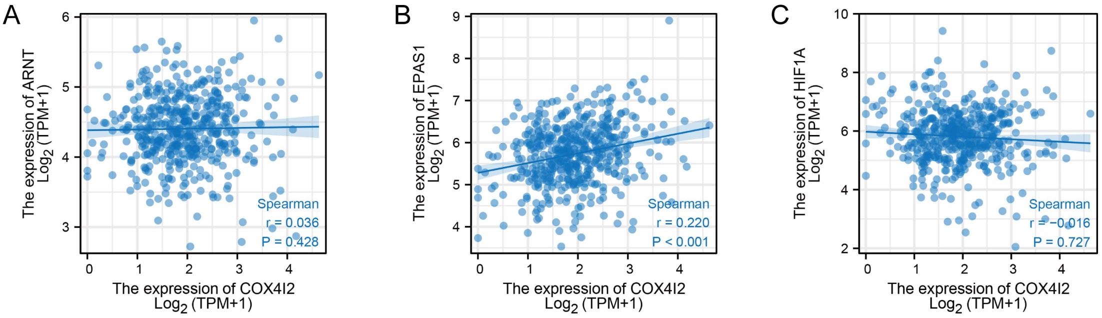

Concentration screening for PD-166866

PD-166866 is an FGF1-selective inhibitor that is often used to study the FGF1 signaling pathway. The compound was dissolved to 10 mM in DMSO stored at −20°C. A CCK-8 kit was used to measure cell viability. Cells were seeded at 4000 cells/well in 96-well plates in 200 μL fresh growth medium containing 10% FBS and were then treated with different concentrations of PD-166866 for 24 h at 37 °C.

At specific times, CCK-8 (10 μL per 100 μL medium) was added to each well, and the cells were incubated for 2 h at 37°C.

Absorbances at 450 nm were read in a microplate reader. Assays were conducted in triplicate.

Fig. S2. Addition of FGF1 (10ng/mL) rescue COX4I2 knockdown CRC cells EMT phenotype.

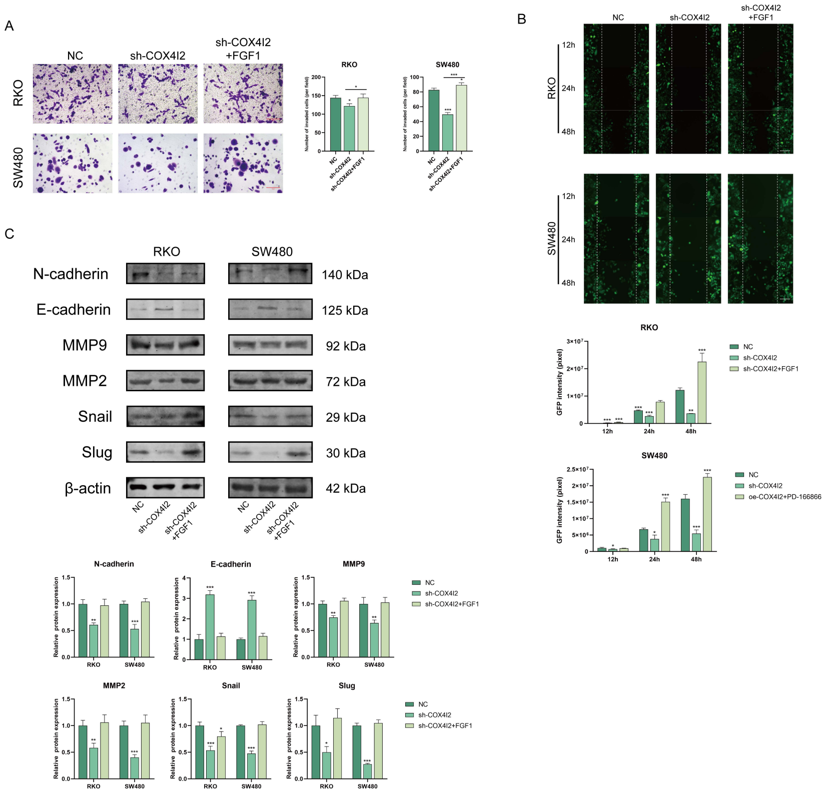

(A) Cell invasion (Transwell assay) (Magnification, ×200, scale bars = 50 μm) (n = 3 replicates).

(B) Cell migration (scratch assay) (Magnification, ×200, scale bars = 50 μm) (n = 3 replicates).

(C) EMT-related proteins shown by western blotting (n = 3 replicates).

Data are expressed as mean± SEM, *P < 0.05; ** P < 0.01; *** P <0.001. All experiments were repeated at least three times, independently.

Fig. S3. Cell proliferation was detected by CCK8 in CRC cells treating with FGF inhibitor

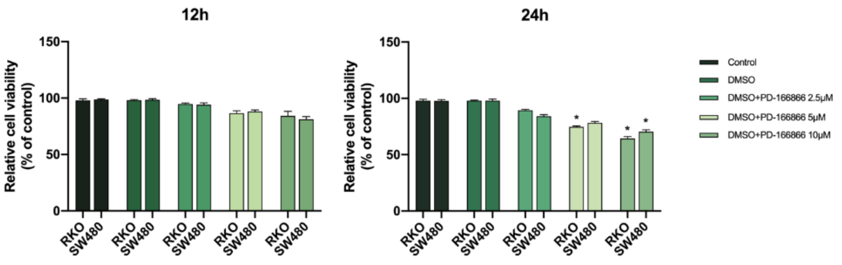

Fig. S4. Evaluation of FGF1 inhibition efficiency

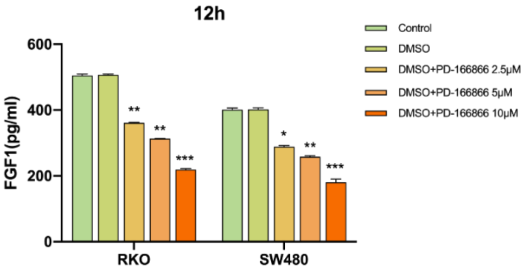

Fig.S5. Analysis of transcription factors of COX4I2-related genes

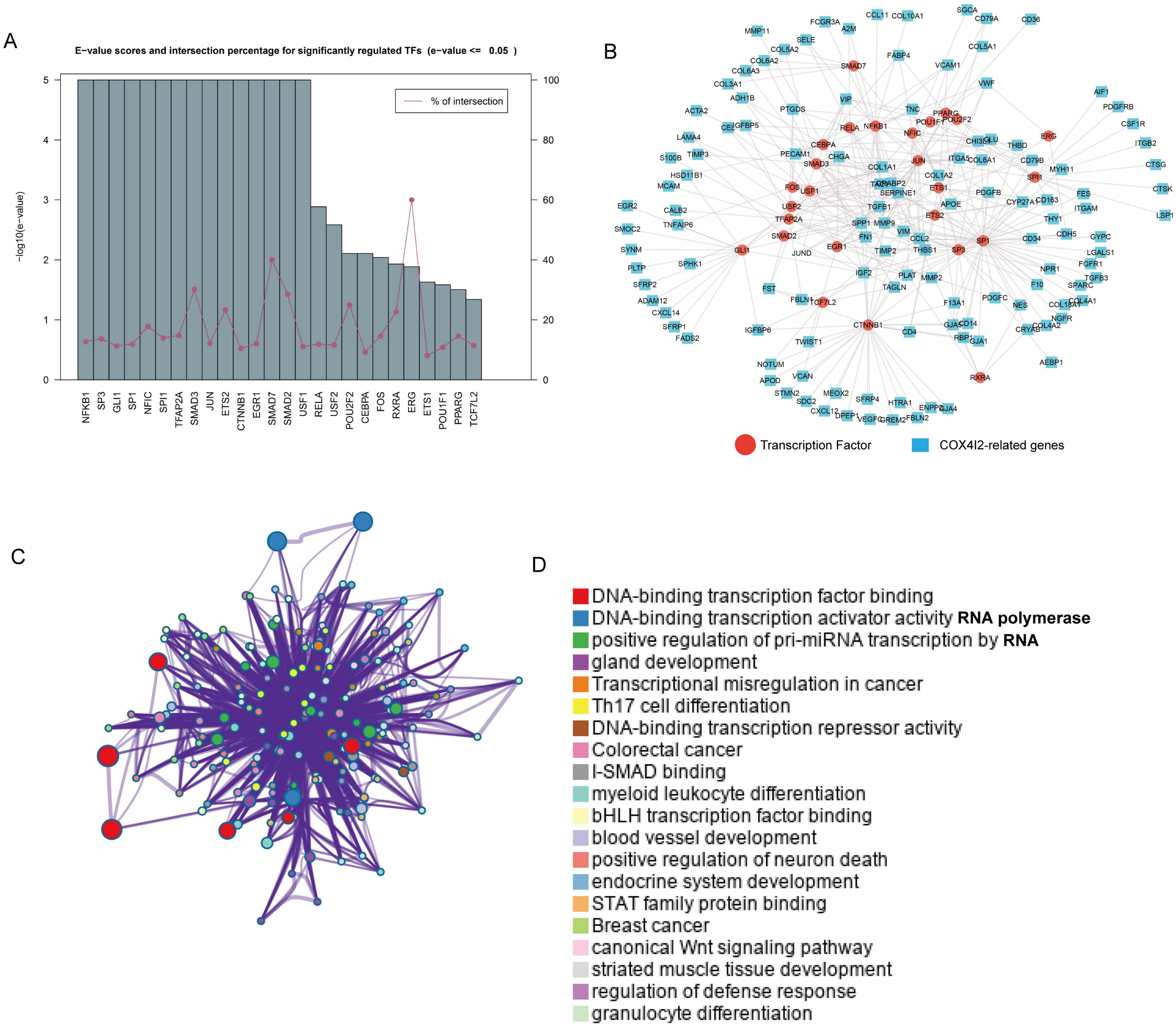

(A) The 26 most transcriptional genes that regulate the number of genes.

(B) Transcription Factor-Gene Network.

(C) Enrichment analysis of transcription factors.

Fig. S6. Additional addition of FGF1 (10ng/mL) rescues the phenotype after knockdown of COX4I2, including angiogenesis and fibroblast activation.

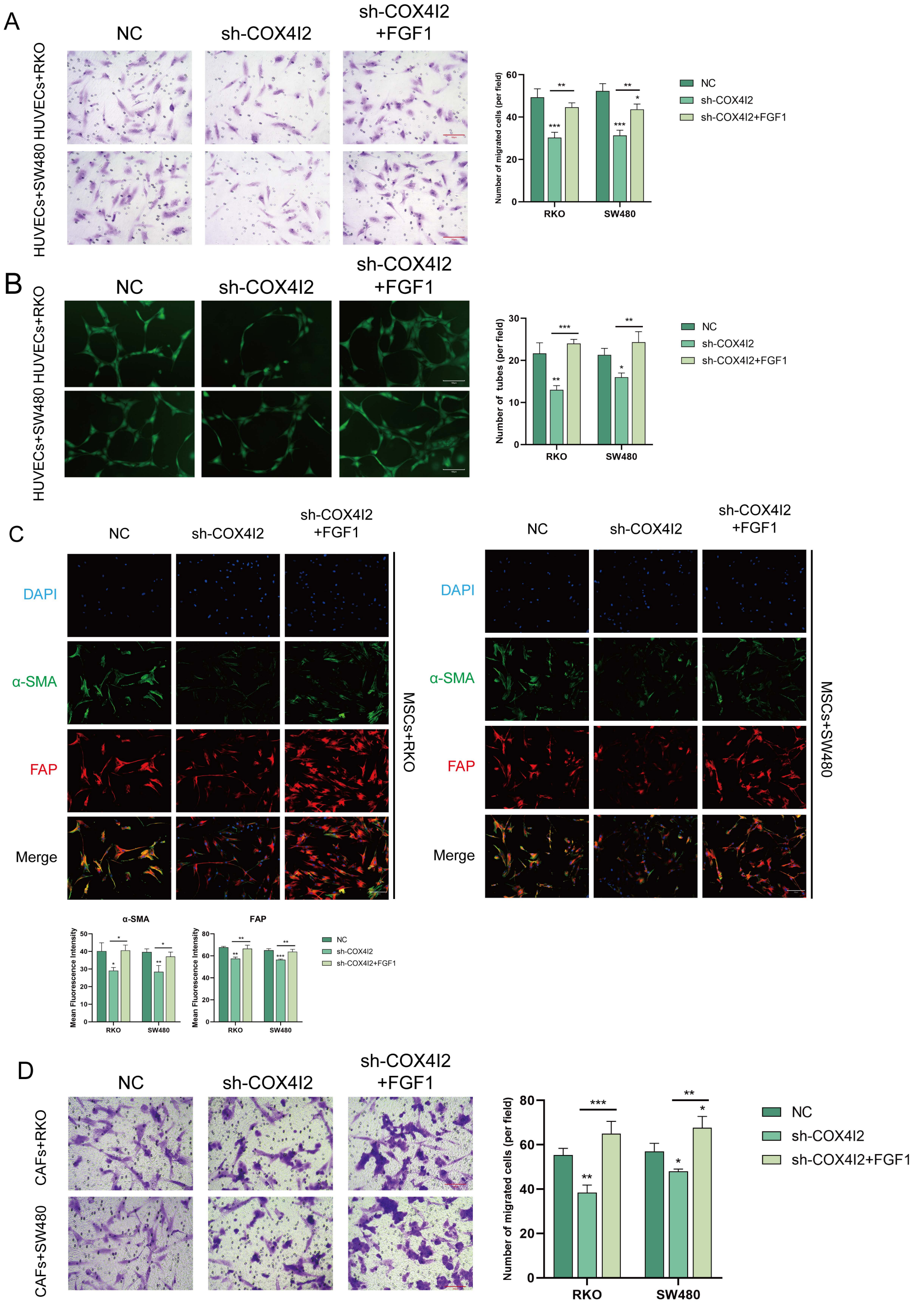

(A) HUVEC migration analysis (Transwell assay) (Magnification, ×200, scale bars = 50 μm) (n = 3 replicates).

(B) The tube formation assay (Magnification, ×400, scale bars = 20 μm) (n = 3 replicates).

(C)Immunofluorescence of MSCs co-cultured with CRC cells (Magnification, ×400, scale bars = 20 μm) (n = 3 replicates).

(D) Recruitment of CAFs by CRC cells (Transwell assay) (Magnification, ×200, scale bars = 50 μm) (n = 3 replicates). Cell numbers assessed by Image J.

Data are expressed as mean± SEM, *P < 0.05; ** P < 0.01; *** P <0.001. All experiments were repeated at least three times, independently.
